# Crossing Pathological Boundaries: Multi-Target Restoration of the Neurovascular Unit in Alzheimer's and Vascular Dementia—From Modern Therapeutics to Traditional Chinese Medicine

**DOI:** 10.14336/AD.2025.0801

**Published:** 2026-08-23

**Authors:** Tengyu Zhao, Pengyu Pan, Ying Jia, Yuhan Zhou, Xinyue Zhang, Huibo Guan, Quan Li, Yanyan Zhou

**Affiliations:** ^1^School of Basic Medicine, Heilongjiang University of Chinese Medicine, Harbin, 150040, China.; ^2^Second Affiliated Hospital of Heilongjiang University of Chinese Medicine, Harbin, 150040, China.; ^3^Key Laboratory of Basic Theory Research on Traditional Chinese Medicine, Harbin, 150040, China.

**Keywords:** Neurovascular unit, Alzheimer’s disease;, Vascular dementia, Multi-target therapy, Traditional Chinese medicine, Blood-brain barrier, Neurovascular coupling

## Abstract

Alzheimer's disease (AD) and vascular dementia (VD) are the two most common forms of dementia, and they share common mechanisms, especially in regard to neurovascular dysfunction. There has been increasing evidence that the disruption of the neurovascular unit (NVU), which consists of endothelial cells, pericytes, astrocytes, microglia, neurons, and basement membrane, is one of the key early events in both AD and VD. The objective of this review is to summarize the structure and physiological function of the NVU, then discuss the pathological remodeling of the NVU in AD and VD and finally, show emerging evidence of multi-target approaches that restore the NVU and neurovascular protection. We begin with a description of the structure, and dietary regulatory roles of the NVU in cerebral homeostasis, especially related to Aβ, the blood-brain barrier (BBB), and neurovascular coupling (NVC). The NVU is then related to the pathological events that cause AD and VD, specifically to impaired Aβ clearance, inflammatory cascades, oxidative stress, and neurovascular uncoupling. Finally, the discussion focuses on a multi-target approach involving exercise, estrogen therapy, mesenchymal stem cells/exosomes, remote ischemic conditioning (RIC), and mindfulness meditation, and analyzes its implications for recovering NVU structure and function. We also discuss the concept of traditional Chinese medicine (TCM) approaches associated with NVU modulation with herbal formulas, traditional Chinese exercises and acupuncture, which has integrative pathways for MVU modulation. NVU dysfunction has a significant and converging impact on the development of both AD and VD. There is considerable support for multi-pathway neurovascular unit targeting, which should show a significant delay in cognitive decline. Incorporating multi-modal evidence from contemporary and traditional medical systems could offer new insights for individualized, neurovascular-targeted therapy for dementia.

## 1. Introduction

Alzheimer's disease (AD) represents the most prevalent cause of dementia seen in the elderly. Pathologically, AD is characterized by an extracellular deposition of amyloid-beta (Aβ) plaques, and intracellular aggregation of neurofibrillary tangles due to hyperphosphorylated tau, which results in progressive neuronal dysfunction and ultimately neuronal death [1]. In contrast, vascular dementia (VD) mainly arises from cerebrovascular disease, often compounded by cardiovascular risk factors [2]. AD and VD represent the two most common forms of dementia and clinically, their pathologies will often overlap to a greater degree than conventionally anticipated, with the mixed type dementia being the most commonly diagnosed type of dementia in clinical practice [3]. In fact, AD and VD share interrelated and mutually reinforcing pathogenic mechanisms. Midlife risk factors such as hypertension, obesity, smoking, physical inactivity, atherosclerosis, and diabetes significantly increase the likelihood of both AD and VD [4]. Notably, the Lancet Commission on dementia prevention, intervention, and care summary for 2020 states modifying these risk factors could prevent up to 40% of dementia cases [5].

Cerebrovascular changes have been characterized as well, including microcirculatory pathology, neuro-vascular unit (NVU) failure, hypercoagulable states, and hypertensive vascular remodeling, among others in AD [6]. In some cases, impaired cerebrovascular function and reduced cerebral blood flow (CBF) occur even before the onset of significant Aβ accumulation [7]. Furthermore, decreased CBF and glucose metabolism have been observed in AD patients, APP transgenic mouse models and APOE4 carriers. This indicates that cerebrovascular dysfunction may represent a functional contributor to the risk of AD [8]. These observations demonstrate the shared and convergent mechanisms between AD and VD.

**Figure 1.**
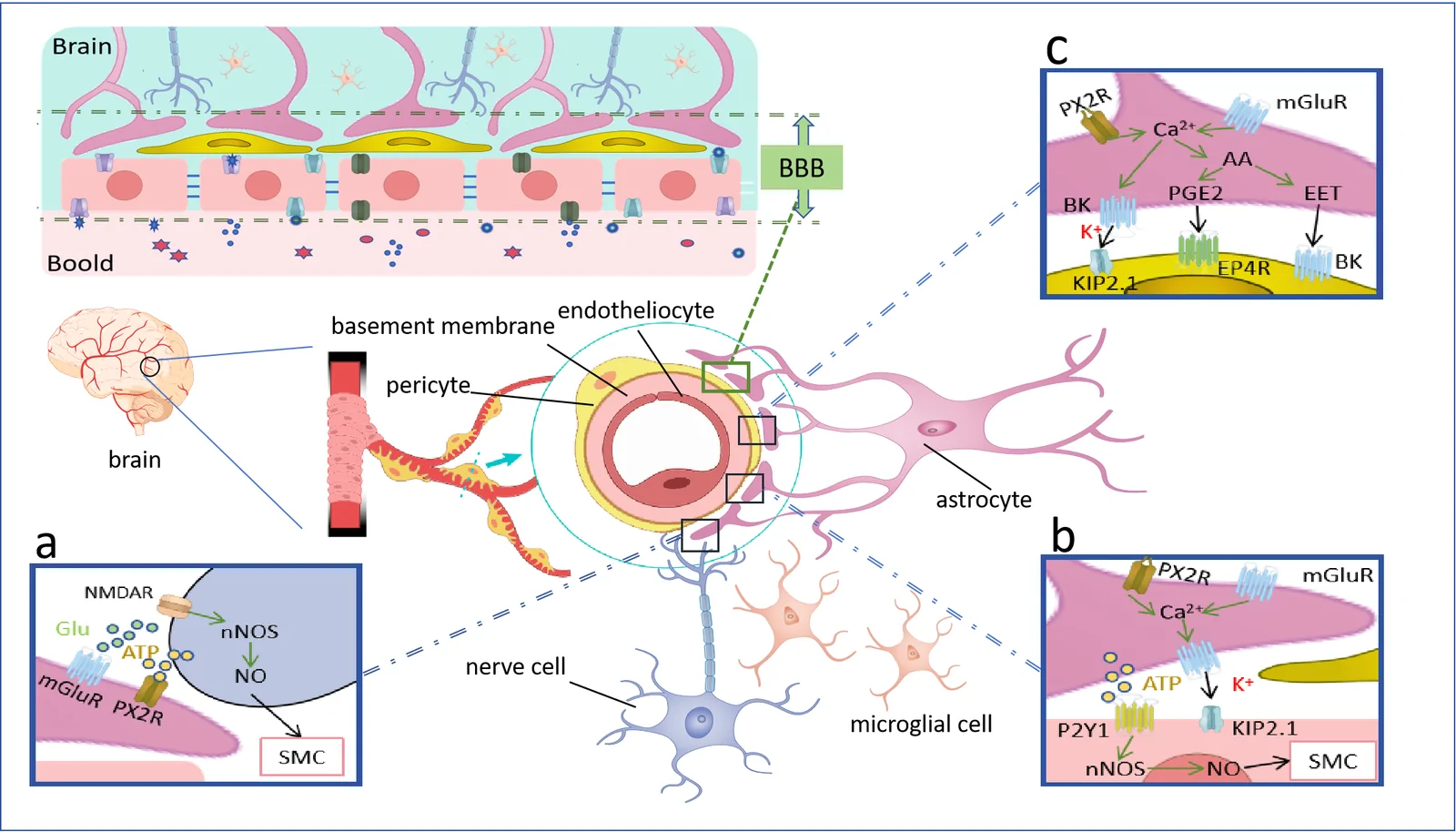
**The structure and function of the neurovascular unit**. The NVU is a key structural and functional component of the brain parenchyma, comprising cerebral microvascular endothelial cells, pericytes, vascular smooth muscle cells, astrocytes, microglia, neurons, and the surrounding basement membrane and extracellular matrix. Under physiological conditions, the NVU maintains brain homeostasis primarily through two mechanisms: BBB and NVC. The BBB is formed by tightly connected endothelial cells, pericytes, astrocytic endfeet, the basement membrane, and associated extracellular matrix components. It serves as a critical interface between the bloodstream and the brain, preventing the entry of blood cells, pathogens, and neurotoxic plasma components into the brain parenchyma. NVC dynamically regulates cerebral microcirculation to match local blood flow with neuronal demand, thereby ensuring optimal cerebral perfusion and metabolic support. Its mechanisms include: First, neuronal activity triggers calcium influx in astrocytes, leading to the release of vasodilators such as NO, EETs, and PGE_2_, which act on smooth muscle cells to induce arteriolar dilation. Second, neuronal depolarization opens voltage-gated calcium channels, promoting Ca²^+^ influx and activation of neuronal nNOS, thereby increasing NO production and facilitating endothelial relaxation through NO signaling or caveolin-dependent pathways. Third, neuronal activation and Ca²^+^-dependent K^+^ efflux from microglial endfeet activate Kir2.1 channels on endothelial cells, contributing to local capillary vasodilation.

The NVU, composed of neurons, glia, endothelial cells, pericytes, and extracellular matrix, plays a pivotal role in maintaining brain homeostasis and regulating precise blood flow in response to neural activity. Recent studies have shown NVU dysfunction to be a common pathological entity across neurological diseases, particularly neurodegenerative and cerebrovascular diseases [9]. With an emergent view of NVC, a shift in perspective of dementia pathogenesis is now informed by NVU architecture and given evidence of NVU dysfunction in both AD and VD, has also emphasized the unique opportunity for development of multi-dimensional interventions aimed at NVU function. Both AD and VD share disruptions in NVU integrity, including compromised blood-brain barrier (BBB) function and impaired NVC, further reinforcing the significance of NVU in the progression of dementia [10-11].

## 2. The concept of the neurovascular unit

The NVU is a functional and structural ensemble built from the central nervous and vascular systems of the brain. It accommodates the high metabolic needs of the brain, facilitates rapid changes in regional cerebral blood flow in accordance with neuronal activity, and delicately regulates the transport of substances within blood and brain tissue [12]. The NVU consists of cerebral microvascular endothelial cells, pericytes, vascular smooth muscle cells, glial cells (including astrocytes, oligodendrocytes, and microglia), neurons, as well as the basement membrane and extracellular matrix components within the cerebral vasculature [13-14]. Each cellular and non-cellular component communicate at a high level of complexity to modulate vasomotor tone, regulate neurotransmitter signalling, and facilitate molecular transport across the vascular interface that upholds cerebral microcirculatory homeostasis and functional stability [15-16] (Fig. 1).

The NVU maintains brain homeostasis through two primary regulatory systems: the BBB and NVC. The BBB consists of endothelial cells that are joined by pericytes, astrocyte endfeet, basement membranes, extracellular matrix (ECM). The BBB acts as a composite physical barrier and biochemical barrier that actively regulates what blood-derived substances can enter the brain and facilitates the clearance of metabolic byproducts [17]. Brain microvascular endothelial cells form a continuous monolayer joined together by tight and adherens junctions, constituting the structural core of the BBB. These cells demonstrate very low levels of transcytosis and vesicle-mediated solute movement and rely on polarized transport mechanisms at the luminal and abluminal membranes to regulate molecular influx and efflux [18]. Pericytes within the basement membrane make physical contact with endothelial cells using N-cadherin and connexins [19-20]. They also secrete factors such as TGF-β, ANG-1, and vascular endothelial growth factor (VEGF) to enhance the expression of junction proteins like occludin, thus modulating BBB permeability [21]. Pericytes modulate BBB structure and function by influencing the polarization and distribution of astrocytic endfeet [22]. Astrocytic endfeet, which are found over the CNS capillaries, are important in secreting neurotrophic and extracellular matrix proteins that are necessary for BBB development, maintenance, and repair [23]. These endfeet also provide a structural coverage that participates in regulation of the barrier's permeability [24]. Finally, the basement membrane, a specific kind of ECM, provides mechanical stability and can provide some molecular filtration. The many different ECM components involved (fibronectin, proteoglycans) are important for dynamic cell signaling and communication in order to sustain the structure and associated functions of the barrier [25-26].

NVC is a dynamic link between neuronal activity and local CBF to ensure timely delivery of oxygen and nutrients to activated brain regions and the removal of waste by-products. Unlike baseline CBF regulation, NVC is due to tightly coupled interactions among neurons, astrocytes, endothelial cells, pericytes, and smooth muscle cells [27-28]. Although some mechanisms remain incompletely understood, astrocytes are widely believed to serve as key intermediaries that transduce neuronal signals into vascular responses [29]. The current hypothesis is that NVC occurs via three primary pathways. First, neuronal activity leads to calcium entry in astrocytes which leads to the release of various vasodilating factors such as nitric oxide (NO), epoxyeicosatrienoic acids (EETs), and prostaglandin E2 (PGE2). These molecules act on smooth muscle cells to induce arteriolar dilation. Second, neuronal depolarization leads to the opening of voltage-gated calcium channels which allow Ca2^+^ entry and activation of neuronal nitric oxide synthase (nNOS) that increases production of NO and relaxes the endothelium via NO-signaling and/or caveolin dependent process. Third, neuronal activity and Ca2^+^ dependent K^+^ efflux from microglial endfeet activate inward-rectifying potassium channels (Kir2.1) on endothelial cells that trigger local capillary dilation [30-34]. The redundancy among these pathways provides compensatory potential so that if one pathway is inhibited the other pathways can be stimulated to preserve perfusion [35]. However, feedback loops and crosstalk among these pathways are not completely understood, so further studies are required to clarify the regulatory network and formulate strategies to alleviate neurovascular dysfunction in pathological states.

## 3. Adaptive Regulation of the NVU in Early Cognitive Impairment

**Figure 2.**
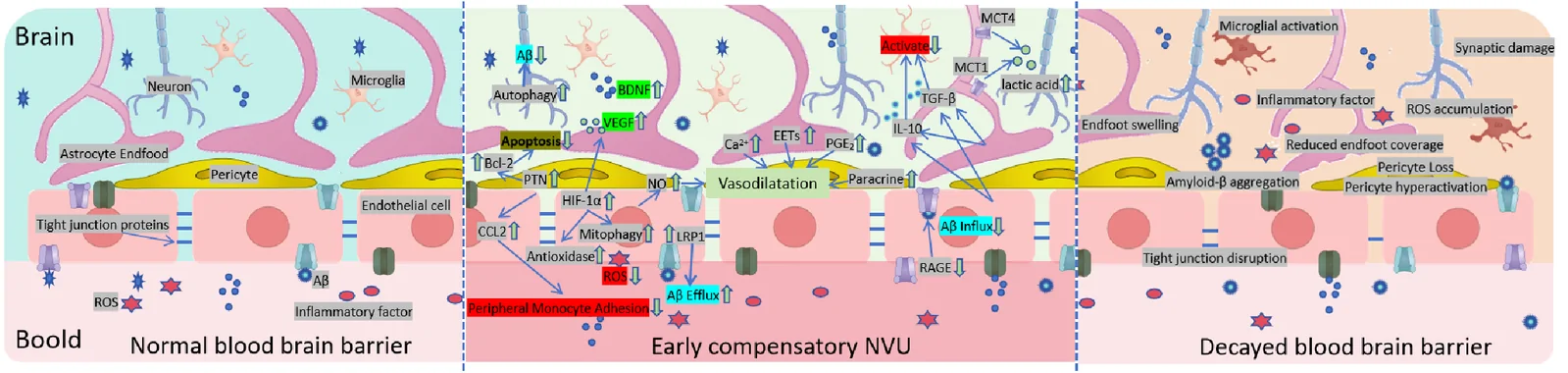
**Changes in the decayed neurovascular unit**. Degeneration of the BBB involves the degradation of tight junction proteins, dysfunction of molecular transporters and vesicular endocytosis, mislocalization of astrocytic endfeet, pericyte loss, and aberrant activation of microglia. The complex interactions and synergistic effects among these changes ultimately lead to increased BBB permeability. In parallel, NVC dysfunction involves a cascade of pathological alterations, including compromised ion exchange and disrupted intercellular signaling among microglia, pericytes, neurons, and endothelial cells. These impairments collectively lead to deficient CBF regulation in response to neuronal activity and metabolic demands. Furthermore, diminished contractile capacity and impaired vasoactive responsiveness in both endothelial cells and pericytes significantly aggravate neurovascular uncoupling, creating a vicious cycle of cerebrovascular dysregulation.

The NVU not only regulates local cerebral blood flow and energy, but it also acts as a "gatekeeper" and "regulator" of BBB integrity and neuroinflammation and mediates brain repair (Fig. 2). It is through the adaptive capabilities of the NVU that we see active compensatory responses to counteract early dysfunction. Functional MRI and PET scanning during the early phases of cognitive impairment have been shown to cause compensatory hyperactivity in specific cortical regions that might include structural or spatial ranked NVU remodeling [36-37].

Mild cognitive impairment (MCI) is the clinical transitional phase between normal cognitive decline with aging and dementia, in which deficits are observed in memory, language, or executive function, but do not significantly impede daily life. During the earliest declines in cognitive performance such as MCI, both VEGF levels and brain-derived neurotrophic factor (BDNF) are transiently elevated [38-39]. The increased levels are understood to be compensatory, in which angiogenesis and synaptic remodeling occurs to maintain a supportive microenvironment for neurons to survive and conduct appropriate function.

During the early stages of Aβ accumulation, neurons activate the autophagy-lysosome system to intracellular Aβ and its precursors (for example, APP C-terminal fragments). The process relies on the inhibition of the mTOR pathway (e.g. AMPK activation), and upregulation of associated autophagy proteins (LC3-II and Beclin-1) that clear toxic Aβ oligomers and inhibit aggregation [40]. Pericytes also contribute to early Aβ clearance by regulating transvascular transport through receptors such as the low-density lipoprotein receptor-related protein 1 (LRP1) and RAGE [41]. Endothelial cells also can promote Aβ efflux from brain to blood via LRP1, whilst inhibiting influx via RAGE, which can affect Aβ accumulation [42]. Astrocytes and microglia likewise become activated early, contributing to Aβ phagocytosis and mediating immune clearance of Aβ [43].

During cerebral hypoperfusion, endothelial cells upregulate NO production to increase cerebral blood flow [44]. Astrocytes differentially respond to neuronal activity through calcium signalling, and promote local capillary dilation, during periods of limited perfusion [45]. Metabolic byproducts (e.g. lactate, H^+^) that accumulate in periods of low-flow induce astrocytic calcium signalling, which can promote the release of local vasoactive mediators (e.g. PGE_2_, EETs), promoting local vasodilation to be as great as possible. Astrocytic signaling may also exert paracrine effects on pericytes at precapillary regions, promoting direct capillary dilation independent of upstream arteriole mechanisms [35].

Disrupted energy metabolism is a hallmark of cognitive impairment, leading to excessive production of reactive oxygen species (ROS), which damage mitochondria and exacerbate cellular stress in a vicious cycle. Under hypoxic or metabolic stress, endothelial cells are reported to upregulate hypoxia-inducible factor-1α (HIF-1α), which drives expression of vascular endothelial growth factor (VEGF) and endothelial nitric oxide synthase (eNOS)-dependent NO production. HIF-1α also drives the expression of BNIP3, which directly activates Drp1-mediated mitochondrial fission and increases PINK1/Parkin-dependent mitophagy. HIF-1α also upregulates antioxidant enzymes such as SOD2 and suppresses mitochondrial ROS production via NO signaling, thereby restoring metabolic homeostasis [46-47]. In early oxidative stress, endothelial cells activate the Nrf2 pathway, leading to increased expression of antioxidant enzymes including SOD, HO-1, and NQO1, which protect blood-brain barrier integrity [48]. Furthermore, astrocytes enhance neuronal metabolism with the astrocyte-neuron lactate shuttle, moving lactate between astrocytes and neurons via MCT1 and MCT4 transporters [49].

Neuroinflammation is a key driver of cognitive impairment. An intact BBB limits peripheral proinflammatory mediators (e.g., IL-6, TNF-α) and immune cell infiltration, thereby suppressing the amplification of central inflammation [50]. In early cognitive dysfunction, astrocytes and endothelial cells in the NVU release anti-inflammatory cytokines such as TGF-β and IL-10 [51]. These factors inhibit overactivation of microglia, particularly by inhibiting the NF-κB/NLRP3 inflammasome pathway, and thus help maintain immune homeostasis in the brain [51]. There are suggestive properties for pericytes as well as a secretion of pleiotrophin (PTN) which causes upregulation of PI3K/Akt signalling reducing the expression of apoptotic proteins (e.g. Bcl-2), in addition to inhibiting caspase-3 dependent cell death and inhibiting/reducing chemokine CCL2, which limits peripheral monocyte recruitment and neuroinflammation [52].

**Figure 3.**
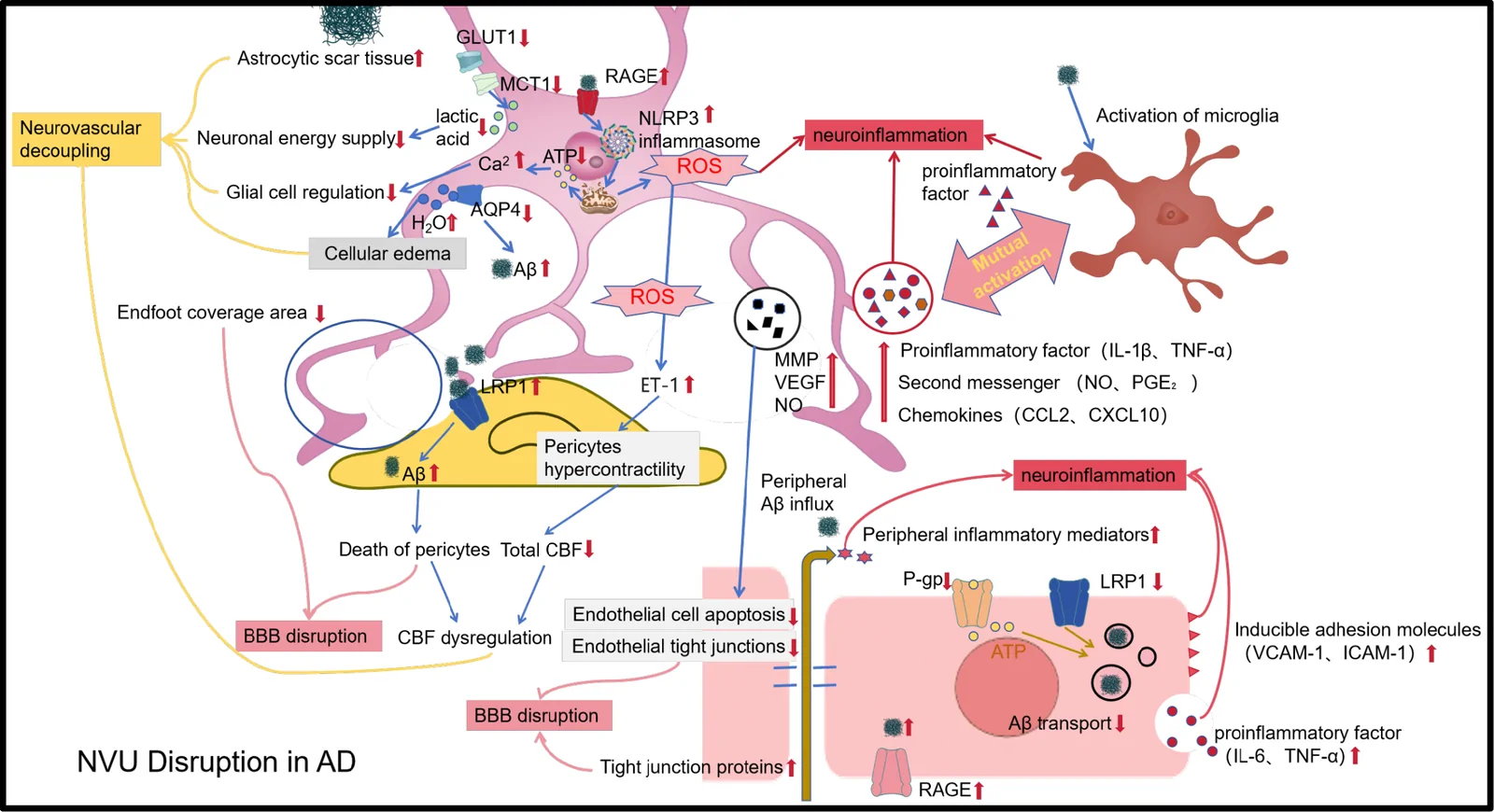
**NUV disruption mechanism in AD**. Although many intercellular interactions within the neurovascular unit (NVU) remain incompletely understood, current evidence suggests that NVU components—including brain microvascular endothelial cells (BMECs), pericytes, astrocytes, and microglia—undergo pathological changes not in isolation, but through complex cross-talk that forms a self-amplifying vicious cycle driving disease progression in AD. First, impaired astrocytic signaling, reduced astrocyte endfoot coupling, and abnormal pericyte activation collectively lead to neurovascular decoupling. These changes coincide with structural alterations such as astrocytic scar formation, decreased endfoot coverage, extracellular matrix remodeling, and pericyte loss. Second, dysfunction of the glymphatic system contributes to water homeostasis imbalance. Coupled with disrupted astrocyte-neuron lactate shuttling and astrocytic mitochondrial dysfunction, this results in a triad of crises: ionic imbalance, energy deficit, and mitochondrial failure. Finally, chronic inflammation sustained by co-activation of microglia and astrocytes, infiltration of peripheral immune cells, and positive-feedback loops between BBB leakage and Aβ accumulation further accelerate NVU degradation. Notably, Aβ itself serves as both a pathological by-product and a key driver—it impairs Aβ clearance by endothelial cells and pericytes, induces dysfunctional microglial phagocytosis, increases BBB permeability, and amplifies NVU injury via its intrinsic neurotoxicity.

## 4. NVU Remodeling in Alzheimer's Disease

In recent years, the role of the NVU in the pathogenesis of AD has gained increasing attention. Notably, reductions in CBF and diminished neurovascular responses to neuronal activation have been detected in the early stages of AD, alongside BBB disruption and leakage of neurotoxic substances [53-55]. These findings suggest that NVU dysfunction may be involved in the early pathophysiological development of AD. Moreover, Aβ exhibits potent neurovascular toxicity: it directly damages endothelial cells, pericytes, and astrocyte endfeet, compromising NVU structural integrity. In turn, NVU dysfunction exacerbates impaired Aβ clearance and accelerates pathological accumulation [56-57]. This bidirectional pathological interplay between Aβ and NVU dysfunction is now considered a major driver of progressive cognitive decline in AD (Fig. 3).

### 4.1 Aβ Clearance Impairment and Inflammatory Cascade in Brain Endothelial Cells

Endothelial Cells which are the main structural unit of the BBB, have a primary role in the transport and clearance of Aβ. This function is mediated by multiple dynamic transmembrane transport systems. The receptor for advanced glycation end products (RAGE), is an influx transporter that is expressed on BBB EC and, is significantly upregulated in the RAGE, BBB EC of AD patients and promotes inappropriate peripheral Aβ transport to the brain [58]. Conversely, LRP1 interacts with APOE to mediate Aβ efflux across the BBB, followed by lysosomal degradation or exocytosis [59]. Furthermore, decreased LRP1 receptor expression results in impaired clearance of Aβ. Glycoprotein (P-gp) is also expressed on the luminal cell membrane of endothelial cells and facilitates active Aβ efflux in coordination with LRP1 via ATP-dependent mechanisms. However, Aβ hastens the degradation of P-gp via the ubiquitin-proteasome system and creating a vicious cycle [60]. Endothelial cell dysfunction also contributes to vascular inflammation during aging and neurodegeneration. Aβ activates pro-inflammatory signaling pathways in endothelial cells which leads to increased expression of adhesion molecules (VCAM-1, ICAM-1) and cytokines (e.g., IL-6, TNF-α), leading to further recruitment of immune cells from the peripheral systems into the brain parenchyma. This inflammation cascade may compromise the integrity of the neurovascular unit (NVU), and result in a pathogenic and chronic inflammatory microenvironment [61].

### 4.2 Pericyte Hypercontraction and Aβ Clearance Dysfunction

Pericyte dysfunction has been implicated in both pathological and cognitive deficits in AD and is believed to play a key role in early disease stages [62]. A mechanism that has been proposed is excessive contraction of pericytes, which significantly correlates with Aβ accumulation that in turn contributes to decreased CBF. This pericyte contraction is caused by Aβ-induced ROS production and hence, endothelin-1 (ET-1) release [63]. Furthermore, pericytes have been shown to facilitate Aβ clearance via the LRP1/APOE mechanism. However, when excess intracellular Aβ accumulates, it results in pericyte death leading to a pathological feedback loop that exacerbates disease progression [64-65]. This mechanism is influenced by APOE isoforms. The APOE4 high-risk variant is shown to promote pericyte apoptosis and breakdown of the BBB through activation of the CypA-NF-κB-MMP-9 signaling pathway whereas protective variants of APOE2/3 can inhibit this signaling pathway via LRP9 activation [66-67].

### 4.3 Astrocyte Reactivity, Neurovascular Uncoupling, and Metabolic Dysregulation

Astrocytes play a key role in the NVU dysfunction in AD. Their complex morphology and susceptibility to AD-related genes (e.g., Apoe, Clu, Fermt2) are associated with their roles in synaptic regulation, metabolic support, and inflammatory responses [68]. Reactive astrocytes in AD exhibit hypertrophy and proinflammatory activation, as well as functional heterogeneity over the course of disease [69-71]. Reactive astrocytes cluster around amyloid plaques to form what is called "astrocytic scars," which may physically isolate regions of pathology, all the while blocking neuron-to-vascular signaling [72]. Reactive astrocytes secrete a variety of pro-inflammatory mediators, including cytokines (IL-1β, TNF-α), chemokines (CCL2, CXCL10), secondary messengers (NO, PGE_2_), and ROS. These factors collectively drive chronic neuroinflammation, resulting in BBB disruption, neurovascular uncoupling, and subsequent neuronal dysfunction [73-74]. Moreover, reactive astrocytes also secrete matrix metalloproteinases (MMPs), VEGF, and NO, which disrupt the integrity of the BBB and can lead to endothelial apoptosis and tight junction loss [75-76].

Astrocytic regulation of the glymphatic system and lactate metabolism is also impaired in AD. Specifically, the expression of aquaporin-4 (AQP4) in astrocytic endfeet is reduced in AD [77]. AQP4 also promotes the efficiency of cerebrospinal fluid-interstitial fluid (CSF-ISF) exchange in the glymphatic system and promotes clearance of β-amyloid (Aβ) along the glymphatic pathways while maintaining cerebral water homeostasis [78]. Consequently, as AQP4 is down-regulated, vascular Aβ deposition occurs, which can lead to BBB breakdown and alterations of water transport, further leading to delays in neurovascular coupling responses [77,79]. Under typical circumstances, astrocytes metabolize glycogen and glucose to produce lactate which is enabled to be shuttled to the neurons via monocarboxylate transporters (MCT1/MCT4) enabling neuronal metabolism to remain active. However, in AD, GLUT1 and MCT1 were downregulated significantly leading to impaired lactate delivery, reduced glucose metabolism, and further neurovascular uncoupling [80-81].

Additionally Aβ-RAGE interactions activate the NLRP3 inflammasome in astrocytes leading to mitochondrial ROS production, ATP depletion and mitochondrial injury [82]. Together, these interactions inhibit astrocytic neurotransmitter uptake (especially glutamate and ATP), increase intracellular calcium and impair communication between the NVU [83]. Moreover, the reduced coverage of blood vessels by activated astrocytic endfeet further impairs neurovascular coupling efficiency [84].

### 4.4 Microglia-Astrocyte Crosstalk and Inflammatory Feedback Loops

Microglia attempt to initially protect against Aβ through phagocytosis and enzymatic degradation (e.g., neprilysin, IDE, ECE) but eventually become chronically activated in AD [85]. Constant exposure of microglia to Aβ results in the activation of the NLRP3 inflammasome through phagocytosis of Aβ, contributing to more Aβ aggregation and inflammation [86]. Proinflammatory cytokines released by microglia activate reactive astrocytes, leading to reactive astrocyte neurotoxicity and damage to the BBB [87]. Reactive astrocytes also release pro-inflammatory mediators (e.g., IL-6, PGE_2_), further activating the inflammatory cascade and promoting further microglial pro-inflammatory polarization. This reciprocal activation creates a chronic feed-forward inflammatory loop, causing further dysfunction of the NVU and resulting in additional cognitive decline [88-89].

## 5. NVU Remodeling in VD

**Figure 4.**
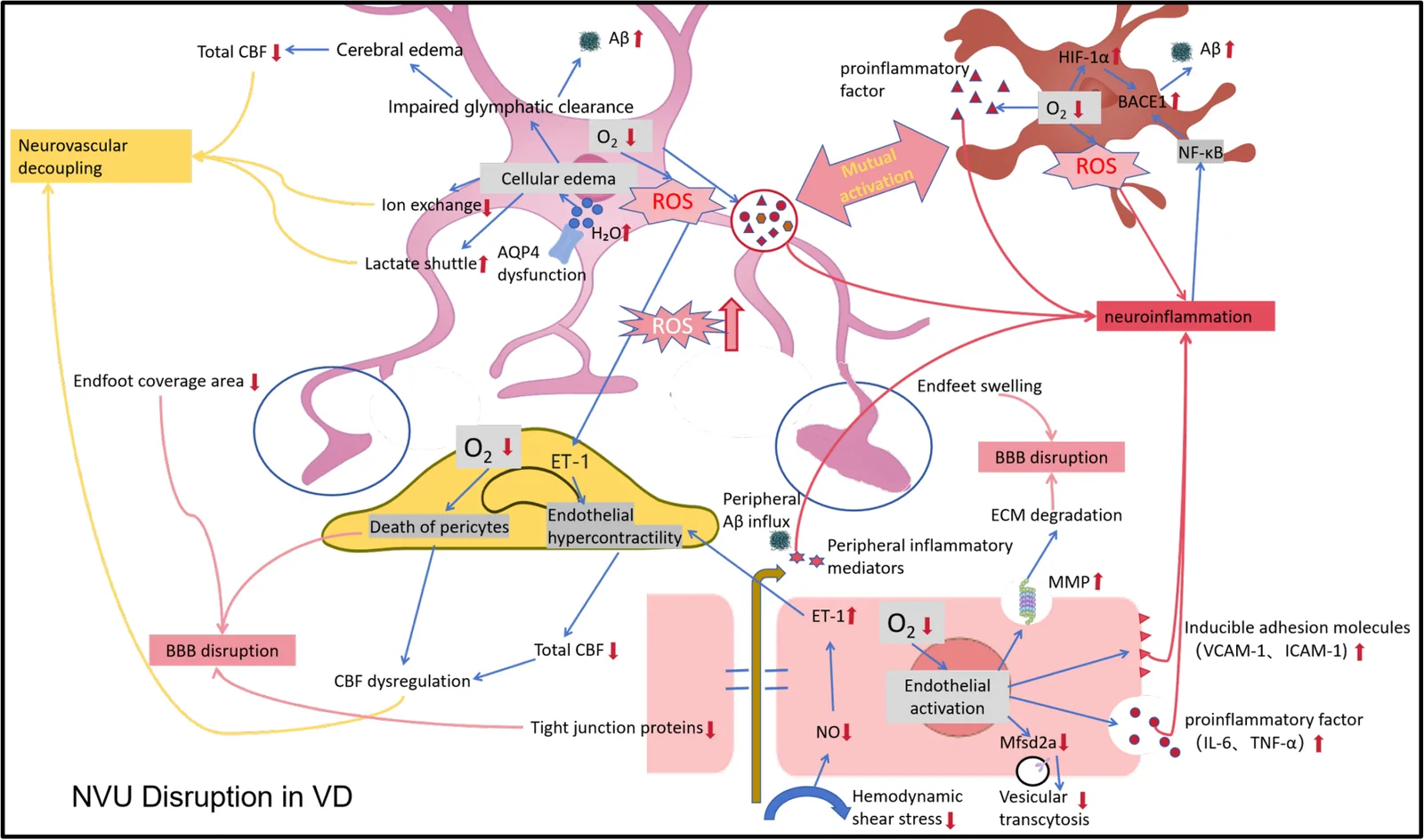
**NUV disruption mechanism in VD**. Although NVU changes in VD have been less extensively studied, available evidence indicates that many of the pathological features parallel those observed in AD. These include loss of astrocytic endfoot coverage, excessive pericyte apoptosis, endothelial transport dysfunction, and tight junction protein degradation—all contributing to BBB breakdown. Moreover, astrocytic swelling, cerebral edema, exaggerated pericyte contraction, and endothelial activation collectively impair cerebral blood flow (CBF) regulation. Inflammatory synergy between astrocytes and microglia, as well as peripheral immune activation triggered by endothelial dysfunction, further exacerbate vascular injury. These pathological processes—driven by chronic hypoxia, mitochondrial dysfunction, and oxidative stress—form a vicious cycle leading to progressive NVU failure. Importantly, excessive Aβ production and deposition may also occur during this process, further linking VD to AD-like neurovascular pathology.

VD is made up of many variants - these include: multi - infarct dementia, small vessel dementia, strategic infarct dementia, hypoperfusion dementia, hemorrhagic dementia, hereditary vascular dementia, and AD with cardiovascular disease - differing primarily in the severity of CBF deprivation [2]. Although these subtypes follow different pathological trajectories, they share common mechanisms, particularly chronic cerebrovascular insufficiency. It is worth noting that CBF reduction due to vascular structural changes is probably the best-known pathological hallmark of VD. However, therapeutic strategies aimed solely at increasing perfusion have yielded inconsistent outcomes [90-91], and standalone antioxidant or anti-inflammatory interventions treatments have not successfully corrected the damage [92-93]. The current evidence would suggest that while an individual insult may initiate VD, a cascade of intertwined, multifactorial pathways drives its progression. Furthermore, larger cohort studies have also established that most cases of VD are related to subcortical small vessel disease, as opposed to large cortical infarcts [94], confirming that chronic pathological changes are more consequential to VD than acute infarction (Fig. 4).

### 5.1 NVU Dysfunction Induced by Chronic Cerebral Hypoperfusion (CCH)

Regardless of subtype, chronic cerebral hypoperfusion (CCH) is a common pathological basis for VD, characterized by persistently reduced cerebral blood flow and insufficient oxygen and glucose supply. Prolonged CBF reduction leads to a widespread neuropathological changes. Under such conditions, the brain does not have sufficient levels of oxygen and glucose, the energy deficiency results in mitochondrial dysfunction and oxidative stress, ATP depletion, neuro-inflammation, and ultimately cellular death. These processes exacerbate one another in a vicious cycle, as extensively reviewed elsewhere [95-97]. The current section will explicate the cellular and molecular alterations in NVU components related to chronic hypoxia. By promoting structural remodelling and injury in the NVU, CCH disrupts NVC and BBB integrity, ultimately diminishing CBF [97-98]. This pathological cascade accelerates VD development.

Endothelial cells respond to hypoxia by upregulating pro-inflammatory molecules, such as intercellular adhesion molecule-1 (ICAM-1), vascular cell adhesion molecule-1 (VCAM-1), and cytokines including IL-6 and TNF-α [99]. These effects lead to peripheral immune cells infiltrating the brain and MMP expression which degrades the ECM and damages the BBB [100]. Additionally, hypoxia lowers expression of Mfsd2a which is a regulator of inhibition of transcytosis in endothelial cells, increasing permeability of the BBB via increased vesicular transport [101]. In VD, tight junction proteins claudin-5 and occludin are considerably downregulated, which likewise decreases endothelial junction density and results in ionic leaking across the BBB [102]. Chronic hypoperfusion lowers shear stress and inhibits endothelial NO synthesis, reducing vasodilation and increasing ET-1 expression, which contributes to continuous vasoconstriction and worsening of cerebral microcirculation [103].

Pericytes are one of the earliest NVU components that are impacted by CCH. Changes in pericyte coverage and call contraction have been shown in the VD models [104-105], preceding structural changes in other components of the BBB. These pericyte deficits downregulate tight junction proteins via disruption of TGF-β/PDGFRβ signaling and diminish endothelial support, leading to increased BBB leakage [106].

Astrocytes are also subject to significant changes in cases of chronic hypoxia. CBF insufficiency results in astrocytic swelling originating from dysfunction of aquaporins, such as AQP4 and AQP9 [107]. Hypoxia also induces BBB disruption and vasogenic edema, leading to intracellular accumulation of water and proteins in astrocytes [108]. With swelling of astrocytes, ionic exchange and glymphatic clearance are impaired, which can affect cerebral water homeostasis, leading to further edema, increased intracranial pressure, and continued reductions in CBF [108-109]. In addition, reactive astrocytes in VD can have declining endfoot presence which alters BBB reconstruction and astrocyte-pericyte coupling mechanisms [110]. While there has been little investigation into the changes to monocarboxylate transporters (MCT1/MCT4) in VD, astrocytic swelling and edema likely reduce lactate shuttling, compounding neuronal energy deficits in hypoxic conditions. Similar to AD, astrocyte-microglia crosstalk in VD leads to neuroinflammation and glial reactivity that sustains a chronic inflammatory condition [95].

### 5.2 Aβ Contribution to NVU Disruption in CCH

Despite Aβ pathology primarily being considered an element of AD, underlying evidence suggests that Aβ pathology, similarly, contributes to NVU disruption in VD. CCH induces a variety of insults including a hypoxic and inflammatory microenvironment which drives enhanced Aβ production and aggregation. CCH-induced hypoxia increases the expression of HIF-1α, which enhances transcription and activity of β-site amyloid precursor protein cleaving enzyme 1 (BACE1) [111]. Hypoxia inhibits prolyl hydroxylases (PHDs), leading to the stabilization and accumulation of HIF-1α protein. The stabilized HIF-1α protein binds to hypoxia response elements (HREs) located within the promoter of the BACE1 gene, and drives the expression of BACE1 [112]. Conditional knockout of HIF-1α in mouse cortex and hippocampus that demonstrated significantly reduced levels of BACE1 confirms HIF-1α as a major contributor to Aβ generation under hypoxic conditions [113].

CCH is also associated with transcriptional suppression due to the inhibition of the ubiquitin-proteasome system, ultimately to also decrease the degradation of BACE1 and subsequently increase levels of Aβ [114]. Inflammatory mediators (e.g. IL-6 and TNF-α) released from activated microglia and astrocytes due to hypoxia can also signal via NF-κB and increase expression of BACE1 promoter; thus may also increase Aβ production [115]. Furthermore, ischemic injury impairs both enzymatic and physical Aβ clearance pathways: neprilysin and other Aβ-degrading enzymes are downregulated, and BBB leakage and glymphatic dysfunction hinder Aβ efflux [116]. These processes together contribute to pathological Aβ accumulation in VD.

Revisiting the mechanisms of NVU disruption in both AD and VD illustrates considerable parallels. While each disorder is initiated by different pathological triggers, both activate broad, multifactorial cascades in the NVU that eventually lead to similar structural and functional impairment. This overlap also supports the importance of targeting common downstream NVU pathways, regardless of the initiating insult. To illustrate both common and disease-specific NVU remodeling mechanisms with evidence from currently available studies, we constructed a comparison table of pathological characteristics of the NVU in AD and VD. See Table 1 for a detailed comparison of NVU impairment mechanisms in AD and VD. As the pace of research continues to expand, the mechanistic relationships between these pathways will become increasingly interrelated, generating more potential overlap and commonality points.

**Table 1:** Comparative mechanisms of NVU disruption in AD and VD.

NVU Component and Affected Function	Shared Mechanisms	Shared Pathological Outcomes	AD-Specific Mechanisms	VD-Specific Mechanisms
**Endothelial Cells**	Overexpression of adhesion molecules (VCAM-1, ICAM-1) and inflammatory cytokines (IL-6, TNF-α)	Endothelial inflammation and oxidative stress → neuroinflammatory cascade	Primarily driven by Aβ accumulation [62]	Primarily driven by hypoxia-induced inflammation [100]
	Downregulation of tight junction proteins (claudin-5, occludin)	Disruption of tight junctions → BBB breakdown	Mediated by Aβ-induced MMP-9 and VEGF expression [77]	Caused by chronic hypoperfusion and inflammatory MMP release [101,103]
	Not well characterized in AD	Sustained vasoconstriction → NVU uncoupling	Not reported	Chronic cerebral hypoperfusion reduces NO production, increases endothelin-1 expression, impairs vasodilation [104]
	Not well characterized in AD	Increased vesicular transcytosis → BBB disruption	Not reported	Hypoxia increases endothelial transcytosis via Mfsd2a downregulation [102]
**Pericytes**	Increased contraction	Pericyte contraction → BBB breakdown and NVU uncoupling	Aβ-induced ROS enhances ET-1 release leading to pericyte overconstriction [64]	Pericyte hypercontraction observed; potentially driven by elevated ET-1 expression (mechanism under investigation) [105]
	Decreased pericyte coverage	Impaired vascular integrity and NVU uncoupling	Aβ-LRP1/APOE-mediated pericyte death and dropout [67-68]	disrupt TGF-β/PDGFRβ signaling [107]
**Astrocytes**	Reactive astrogliosis and chronic inflammation	End-foot coverage loss → inflammation and NVU uncoupling	Aβ triggers hypertrophy and scar formation, releases cytokines (IL-1β, TNF-α), ROS, chemokines (CCL2, CXCL10) [74-75]	Possibly secondary to microglial activation under hypoxia [111]
	Secretion of VEGF, NO, and MMPs	Endothelial apoptosis, tight junction degradation → BBB damage	Confirmed in Aβ-induced models [76-77]	Likely but not yet confirmed mechanistically [111]
	Dysregulation of AQP4	Astrocytic end-foot swelling → NVU uncoupling	Aβ-related AQP4 mislocalization [78,80]	Hypoxia-linked downregulation of AQP4 and AQP9 [108]
	Impaired GLUT1 and MCT1/4 expression	Decreased lactate shuttle efficiency → NVU uncoupling	Significant reduction in astrocytic GLUT1 and MCT1 expression [81-82]	Not yet fully assessed; cytotoxic edema may hinder lactate diffusion [109-110]
	Not well characterized in VD	NLRP3 inflammasome activation, impaired glutamate and ATP clearance, calcium overload→neuroinflammation and NVU uncoupling	Aβ-RAGE interaction triggers mtROS, calcium dysregulation [83-84]	Not reported
**Microglia**	Activation and crosstalk with astrocytes	Inflammatory cascade → NVU damage	Aβ-mediated chronic activation [89-90]	Hypoxia-induced ROS and inflammation [96].
**Aβ Pathology**		Aβ accumulation → NVU damage amplification	Upregulated RAGE enhances influx; downregulated LRP1 & P-gp impair efflux; Aβ promotes self-retention by downregulating P-gp via ubiquitin-proteasome degradation [59-61]	Hypoxia via HIF-1α increases BACE1 expression and impairs Aβ degradation; inflammation enhances Aβ production [112-117]
	Impaired glymphatic drainage	Impaired Aβ clearance → accumulation and NVU breakdown	Primary mechanism [78]	Resulting from ischemia-induced glymphatic dysfunction [108]

## 6. NVU-Based Reframing of Dementia: From Clinical Evidence to Shared Pathogenesis in AD and VD

As we re-evaluate the two main forms of chronic dementia, AD and slowly progressive VD, from an NVU perspective, we realize that the boundaries between them are far less distinct than traditionally assumed. Recent investigations of NU dysfunction are fundamentally challenging our traditional compartmentalization of AD and VD based exclusively on Aβ pathology or vascular lesions. During the construction of this review, we have increasingly come to the same conclusion—that the NVU may provide a common structural and functional basis of commonality between AD and VD. Consequently, in this section, we will explore the converging mechanisms of AD and VD more deeply, drawing upon recent literature and experimental data to further highlight the unifying potential and critical importance of the NVU in reframing dementia pathogenesis.

### 6.1 Genetic susceptibilities

Emerging data has shown that the most significant genetic risk factors for AD and cerebrovascular disease are closely linked to NVU dysfunction. The ε4 allele of APOE4, which is the most potent genetic risk factor for late-onset AD, directly causes vascular injury and BBB disruption, independent from amyloid or tau pathogenesis [117]. Astrocytic APOE4 selectively induces BBB leakage, likely via MMP9-mediated degradation of vascular basement membrane components [118].

The Notch3 gene, important in CADASIL pathology, may contribute to AD pathogenesis indirectly via promotion of cerebrovascular dysfunction (e.g., BBB leakage, glymphatic impairment, and reduced Aβ clearance), leading to amyloid accumulation [119]. In the AD-associated genetic risk factor SORL1, it has been determined SORL1 can increase vascular Aβ deposition, reduce endothelial function, and reduce clearance of vascular Aβ, consequently damaging the cerebrovasculature. Notably, SORL1 mutation carriers display increased white matter hyperintensities (WMHs), a marker of vascular injury more severe than non-mutation carriers [120]. Similarly, TREM2, identified as a central mediator of immune signaling in neurodegeneration, has shown a pathogenic role in both AD and VD [121]. Importantly, these genetic knockout models for each of the risk genes demonstrate widespread NVU disruptions, further underscoring the unifying role of NVU dysfunction in dementia pathogenesis.

### 6.2 Shared Vascular Risk Factors and Aβ Pathology

Systemic vascular risk factors collectively worsen both AD and VD through their influence on the NVU. Chronic hypertension, diabetes, and metabolic syndrome are consistently overrepresented among individuals with both disorders [122-123]. Hypertension causes cerebral small artery sclerosis, promotes BBB leakage, and enhances Aβ deposition and neuroinflammation. In addition, vasoconstriction associated with hypertension causes chronic cerebral hypoperfusion, which enhances neuronal injury and synaptic dysfunction—key contributors to the development of lacunar infarcts and white matter hyperintensities (WMHs) [124]. Other common metabolic syndrome comorbidities may include insulin resistance, obesity, and dyslipidemia, each of which may occur independently or synergistically to contribute to oxidative stress, endothelial dysfunction, and cerebral hypoperfusion [125]. These vascular/inflammatory processes also contribute to Aβ deposition by driving compensatory upregulation of APP processing toward the amyloidogenic cascade [126]. As addressed in Section 4. 2, vascular injury increases Aβ production, while simultaneously disrupting transport mechanisms utilized for Aβ clearance, thereby accelerating Aβ accumulation in the CNS. In turn, increased Aβ deposition also causes vascular injury, further creating a self-sustaining loop of pathology for both AD and VD. Together, these interactions converge at the NVU and unify the pathogenic processes of AD and VD.

Thus, the NVU serves as a central hub where diverse pathological processes converge and propagate. Rather than operating in isolation, vascular damage, BBB dysfunction, Aβ accumulation, neuroinflammation, microglial activation, and neurovascular uncoupling dynamically influence each other through NVU-mediated cascades. In the early stages, the NVU may be able to buffer and localize the damage, while also importantly amplifying the insult through propagation across the cellular compartments as the damage progresses to an on-going process. This networked behavior helps explain how an isolated insult (e.g., vascular or amyloid-related) can evolve into widespread neurodegeneration.

### 6.3 Combination Therapies Strategies Support the Role of NVU Restoration in Dementia Treatment

Therefore, in both AD and VD, successful therapeutics may not only consist of removing the primary pathogenic driver, but also restoring NVU function and structural integrity. This strategy shifts treatment focus to preserving or rebuilding the NVU's regulatory ability to resist and resolve multifactorial insults, and it has already shown promise in emerging combinatorial treatment approaches.

One of the pioneering studies was a combination treatment consisting of dietary changes, exercise, cognitive training, and monitoring/control of vascular risk factors to sustain and/or improve cognitive performance in elderly patients from the community at increased risk for dementia [127].

The co-administration of cholinesterase inhibitors (ChEIs) and neural stem cell (NSC) transplantation in AD mouse models exhibited a substantial synaptic density and behavioral performance, in combination, compared to both alone, implying an additive effect for NVU remodeling [128].

Also, estrogen is a major multi-site modulator of the NVU [129]. In a small randomized clinical trial, AD women receiving both tacrine and estrogen replacement therapy demonstrated significant improvements in cognition and global clinical status compared to tacrine only or placebo; the most substantial improvement was observed in participants negative for the APOE ε4 allele genotype [130]. Notably, additional pre-clinical data strengthen the possibility of this synergistic response: among ovariectomized rat models with sufficient basal forebrain cholinergic deficits, combination therapy with donepezil and 17β-estradiol (E2) improved memory acquisition while neither agent was efficacious alone; while notable, it was contingent on relative preservation of normal cholinergic function and also confirmed the NVU to be related to response to treatment [131].

These findings together suggest the NVU may prove a key therapeutic target in dementia.

## 7. Currently Promising Multi-Target Strategies for NVU Restoration and Supporting Evidence

The brain's function depends upon a highly integrated, multi-layered regulatory system to meet its very large energy requirements and highly dynamic information processing. Although transient perturbations or localized loss of function are compensated for by neighboring systems, chronic pathological insults can always disrupt these intricate systems eventually. Over time, dysfunction can propagate from localized areas to broader regions via the intricate NVU network.

At this point, approaches targeting a single pathological pathway often cannot restore cerebral homeostasis. At this point, approaches targeting a single pathological pathway often cannot restore cerebral homeostasis. At this point, approaches targeting a single pathological pathway often cannot restore cerebral homeostasis. NVU repair not only restores cerebral homeostasis but also bolsters resistance to cognitive impairment in neurodegenerative and vascular disorders. Therefore, it is imperative to explore and validate multi-target approaches with robust preclinical support and translational potential.

In the subsequent section of this article, we review cutting-edge developments in several multi-dimensional strategies including: exercise, estrogen therapy, mesenchymal stem cell transplantation, remote ischemic conditioning (RIC) and mindfulness-based approaches, with a focus on their roles in restoring NVU structure and function. To facilitate straightforward analysis of these approaches, we have also developed a table summary (Table 2) that outlines each intervention's primary NVU cellular or molecular target, associated signaling mechanisms, stage of development, and reported or intended clinical outcomes.

### 7.1 Exercise

An expanding array of clinical and preclinical evidence suggests that the advantages of physical exercise with respect to age-related cognitive decline are more extensive than previously appreciated, chiefly due to its multi-faceted support of the NVU. A recent meta-analysis of randomized trials has established that controlled physical activity for 12 weeks yields meaningful improvements in global cognition, physical performance, and patient-reported quality of life in people with AD [132]. Potentially, many exercise modalities (aerobic and resistance training) have a capacity to support neuroplasticity [133]. From various perspectives, the positive effects of exercise on NVU integrity have been affirmed. In the 3xTg-AD mouse model, voluntary exercise improved the functionality of neurons, astrocytes, pericytes, and the extracellular matrix. Exercise upregulated pivotal Aβ-clearing proteins including LRP1, neprilysin, matrix MMP-9, and insulin-degrading enzyme (IDE) that led to better Aβ clearance. At the same time, it stabilized the BBB and increased glymphatic clearance, contributing to decreased cerebral Aβ deposits and neurotoxicity [134].

Blood-brain barrier: The exercise influence on the basement membrane is bidirectional. In 3xTg-AD mice, Aβ pathology thickened collagen IV in the basement membrane and interrupted tight junctions, while exercise normalized the membrane thickness and MMP-9 activity [134]. In a model of stroke repair, exercise preserved expression of type V collagen through TNF-α signaling and attenuated excess MMP-9 expression and activity to aid BBB repair [135]. These observations indicate exercise may aid NVU structural repair by modifying the cerebral microenvironment.

**Table 2:** Overview of Current and Emerging Interventions for NVU Restoration in AD and VD.

Intervention Type	Specific Strategy	NVU Target	Modulated Pathways and Outcomes	Development Stage	Comments
**Non-pharmacological**	Physical Exercise (Aerobic, Resistance, HIIT)	Basement membrane	↓ TNF-α → ↑ type V collagen, ↓ MMP-9 → repair of tight junctions and BBB [127]	Preclinical (animal) [127]	Improves NVU function broadly through enhanced blood flow, BBB repair, glia-neuron balance, and anti-inflammation. Future work should clarify how different exercise types affect specific NVU components and explore integration with drug or cognitive therapies for personalized use.
		Astrocytes	↑ AQP4 polarity → enhanced glymphatic flow → ↑ Aβ clearance [128]	Preclinical (animal) [128]	
		Whole NVU	BDNF↑→ Neurotrophic support [129 ] [130 ]; IGF-1↑ IGF1R↑→Neural protection [132 ]	Clinical [129]; Preclinical (animal) [130,132]	
		NSCs, endothelium, astrocytes	↑ NSC proliferation/differentiation → ↑ VEGF, BDNF, IGF-1 → enhanced neurogenesis and angiogenesis [133]	Preclinical (animal) [133]	
		Cerebral vasculature	↑ vessel compliance, ↓ carotid resistance, ↑ CBF, improved memory [135]	Clinical [135]	
		Microvasculature	↑ microvascular density, ↑ CBF [136]	Preclinical (animal) [136]	
		Endothelial cells	↑ MAPK, ↑ eNOS phosphorylation → ↑ NO → vasodilation, ↑ CBF [138]; ↑ HIF-1α → ↑ ET-1, ↑ BNP → vasodilation, ↑ CBF [139]	Preclinical (animal) [138,139]	
		Systemic inflammation	↓ ROS, ↓ TNF-α, ↓ IL-6 → ↓ chronic inflammation [141]; ↓ CRF, ↓ inflammatory biomarkers → ↓ inflammation [142]	Clinical [141,142]	
**Hormonal Therapy**	17β-estradiol (E2), Estrogen Replacement	Neurons	↑ PI3K/Akt → anti-apoptosis, ↑ energy metabolism, ↑ synaptic plasticity → ↑ neuronal survival [156]; ↑ ER, GPER → ↑ Ca²^+^ → ↑ neurovascular coupling [157-158]; ↑ GPER → ↑ ERK → ↓ glutamate toxicity → ↑ neuronal survival [159]	Preclinical (animal) [156-159]	Modulates all NVU elements by supporting neurons, regulating glia, protecting endothelium, and restoring BBB integrity. Further research is needed to determine optimal timing, delivery routes, and safety for clinical translation.
		Astrocytes	↑ PI3K/Akt, MAPK → ↑ TGF-β1/2 → anti-apoptosis, enhanced survival, ↑ neurotrophic factor secretion [160-161]; ↑ intracellular Ca²^+^ → ↑ neurovascular coupling [162]; ↑ GPER → ↑ autophagy, ↓ pro-inflammatory cytokines [163]; ↑ ER → ↑ astrocyte mitochondrial function [164]	Preclinical (animal) [160-164]	
		Microglia	E2, G-1 → M1 to M2 shift → ↓ TNF-α, IL-1β, IL-6; ↑ TGF-β, IGF-1 → ↓ neuroinflammation, ↑ tissue repair [165-167]	Preclinical (animal) [165-167]	
		Endothelial cells	↑ E2 → ↑ ER-PI3K/Akt → ↑ eNOS → ↑ NO → vasodilation [168-170]; ERα-Caveolin-1 complex essential for NO synthesis [171]; ↑ GPER → EGFR transactivation → ↑ ERK, Src, PI3K → endothelial proliferation, ↑ cerebral microcirculation [172-173]; ↓ ICAM-1, ↓ NF-κB → ↓ immune cell adhesion [174-175]	Preclinical (animal&cell) [165-167]	
		Pericytes	ERα/ERβ/GPER-mediated: ↓ TNF-α-induced migration via MAPK/Akt inhibition [176]; ERβ → ↓ miR-638 → inhibit TNF-α-induced migration → protect BBB [177]	Preclinical (cell) [176-177]	
**Cell-based**	MSC transplantation	Neurons	BDNF, NGF, GDNF,HGF,miRNAs↑ → neuronal survival↑, neurogenesis↑, synaptic plasticity↑[170-171]	Preclinical (animal) [170-171]	Shows multi-target effects via secreted growth factors and exosomes, benefiting NVU repair and slowing neurodegeneration. However, clinical efficacy remains limited; future focus lies in improving delivery, cell targeting, and monitoring NVU response in real time.
		Astrocytes	↑ BDNF, GDNF, NGF, VEGF → enhanced astrocytic support [183]	Preclinical (animal) [183]	
		Microglia	M1 to M2 polarization shift → ↑ autophagy, ↑ plaque clearance [181]	Preclinical (animal) [181]	
		Endothelial cells	↑ angiogenesis, vascular repair via VEGF/HGF → ↑ CBF, BBB stabilization [179-180]	Preclinical (animal) [179-180]	
	MSC-derived exosomes (MSC-exo)	Whole NVU	Deliver neurotrophic miRNAs/proteins → replicate MSC function; ↑ synaptic proteins, ↓ Aβ deposition, ↓ inflammation (more effective than MSCs) [184]	Preclinical (animal) [184]	
**Non-pharmacological**	Remote limb ischemia (RIC)	Vascular system	↑ Notch1 → ↑ CBF, ↑ angiogenesis [186]; ↑ eNOS/NO/nitrite → ↑ CBF, ↑ angiogenesis [187-188]; dynamic CBF regulation ↑ [189]	Clinical (observational) [189]; Preclinical (animal) [186-188]	Non-invasive and low-cost, RIC activates systemic protective pathways to improve cerebral perfusion, reduce inflammation, and preserve BBB integrity. Its clinical value is promising, but more work is needed to clarify mechanisms, standardize protocols, and design wearable tools.
		Endothelial cells	↑ FMD via GLP-1 signaling → improved endothelial function [190-191]; ↓ MMP-9 → ↑ Occludin, ↑ Claudin-5 → ↓ BBB permeability [200]	Clinical [190-191]; Preclinical (animal) [200]	
		Neurons	↑ Bcl-2, ↓ apoptosis, ↑ autophagy-related proteins → neuroprotection [192-194]	Preclinical (animal) [192-194]	
		Endothelial cells	↑ FMD via GLP-1 signaling → improved endothelial function [193-194]; ↓ MMP-9 → ↑ Occludin, ↑ Claudin-5 → ↓ BBB permeability [200]	Clinical [193-194]; Preclinical (animal) [200]	
		Whole NVU/ Microenvironment	↓ ROS, ↑ HO-1, ↑ GSH, ↓ MDA, ↑ Nrf2 → antioxidant activity, ↓ neuroinflammation [195-197]; ↓ hsCRP, IL-6, WBCs, platelets → ↓ neuroinflammation [198]; ↓ ICAM-1, VCAM-1, GFAP, IBA-1 → ↓ neuroinflammation, ↑ cognition, ↓ WM damage, ↓ Aβ [199]	Clinical [198]; Preclinical (animal) [195-197, 199]	
**Non-pharmacological**	Mindfulness-based Meditation	DMN, Prefrontal Cortex	↑ rsFC, ↑ cortical thickness, ↑ resting-state CBF → improved NVC and network function [206-207]	Clinical [206-207]	Enhances top-down cognitive control, increases brain connectivity, and may improve neurovascular coupling and metabolic resilience. Key challenges remain in standardizing practice, validating biomarkers, and adapting research methods to human cognition.
		Endothelial cells	↑ eNOS via DNA methylation → vascular adaptation [208-209]	Clinical [208-209]	
		Glial/ Neuroimmune system	↓ inflammatory markers, ↓ glial activation (epigenetic regulation), ↓ IL-6 [210-211]	Clinical [210-211]	
		Whole NVU	↑ BDNF, ↑ ATP turnover, ↑ intracellular pH → ↑ metabolism & cognitive reserve [207, 210]	Clinical [207, 210]	

Glymphatic system: Exercise improves glymphatic function potentially through astrocyte modulation. High-intensity interval training improved AQP4 polarization in astrocytes to promote interstitial fluid clearance and reduce cerebral Aβ burden, thereby lessening AD-like pathology [136].

Neurotrophic support: Aerobic exercise significantly up-regulates BDNF and insulin-like growth factor-1 (IGF-1) in both peripheral and central compartments. Circulating BDNF concentrations have been reported to increase following exercise in humans [137], and the upregulation of hippocampal BDNF has been reported in animal studies [138]. Exercise increases IGF-1 concentration. Exercise also affects the expression of IGF1R and its post-translational modifications (specifically alterations in ubiquitin-like modifications), which provides neuroprotection in AD models [139-140]. Additional studies suggest that endogenous neural stem cells (NSCs) were responsible for the trophic responses. Exercise increased the proliferation and differentiation of NSCs in aged mice [141]. The newly generated NSCs release other factors (e.g., BDNF, VEGF, IGF-1) that promote neuronal survival and synaptic plasticity [142]. They also impact endothelial cells, astrocytes, and pericytes and promote angiogenesis, BBB repair, and glial modulation—this implies a crucial role in NVU remodeling.

CBF: After one year of moderate-to-high intensity aerobic training, CBF improved, carotid stiffness and vascular resistance decreased, and memory improved in the cognitively intact elderly [143]. Animal studies showed prolonged exercise increased microvascular density and perfusion. The effects diminish after cessation of exercise, further supporting the need for habitual exercise [144-145]. All these effects are mediated, in large part, by endothelial effects. Mechanistically, exercise activates endothelial AMPK phosphorylation, which leads to enhanced eNOS activity and NO release, improving endothelium-dependent vasodilation. Shear stress also activates eNOS and increases NO production during exercise [146]. Moreover, exercise increases HIF-1α, which not only upregulates ET-1 and brain natriuretic peptide (BNP) to modulate vasodilation [147], but also directly enhances VEGF transcription and protein synthesis, thereby supporting vascular regulation and BBB stability within the NVU [148].

Anti-inflammatory and antioxidant effects: Endurance training improves antioxidant enzyme activity and decreases chronic inflammation [149]. A cross-sectional study demonstrated that less reported physical activity in older adults was correlated with increases in CRP and other inflammatory markers [150].

In summary, exercise is a viable, non-pharmacological intervention capable of influencing NVU function holistically by modulating cerebral blood flow, BBB integrity, glia-neuron interaction, and the neuroinflammatory context at the molecular, cellular, and systemic level. Future studies could (1) assess the specific effects of varying exercise interventions on distinct NVU components; (2) include functional imaging and biomarker monitoring; (3) establish combined intervention approaches to pharmacological and cognitive interventions. These efforts will enable individualized and clinically relevant exercise prescriptions.

### 7.2 Estrogen Therapy

The clinical use of estrogen has controversy surrounding its therapeutic window and side effects, but with increased evidence suggesting a substantial neuroprotective role in dementia prevention and treatment it should be noted [151]. Elderly women, as well as women that have undergone oophorectomy, have significantly higher risks for dementia outcomes such as cognitive impairment, cardiovascular pathology, AD occurrence, and pathological neurodegenerative biomarkers [152]. Preclinical studies have shown that estrogen exerts broad neuroprotective effects across multiple cellular pathways within the NVU [153]. These effects have been both genomic mediated by nuclear receptors (ERα and ERβ), as well as via non-genomic signal from membrane bound estrogen receptors (mERs, also referred to as GPR30/GPER).

Neuronal Activity and Function:17β-estradiol (E2) exerts neuroprotective effects through both classical genomic regulation of gene expression and rapid, membrane-initiated non-genomic mechanisms. Both genomic and non-genomic actions of E2 are neuroprotective by promoting neuronal survival and limiting pathological neurotoxicity across a variety of experimental models [154]. Of particular relevance, E2 promotes an activated PI3K/Akt pathway that inhibits apoptotic cell death, regulates cellular energy metabolism, promotes neurite outgrowth, facilitates synaptic plasticity, and promotes synthesis and release of neurotransmitters [155]. Furthermore, activation of both ERs and GPER activate intracellular Ca²^+^ rapidly to initiate and sustain the activity of midbrain dopaminergic neurons [156]. GPER signaling further reduces glutamate- or ischemia-induced neurotoxicity via ERK signaling cascades, reinforcing E2's protective effects on neurons [157].

Astrocyte Modulation: E2 modulated astrocyte function through MAPK and PI3K/Akt signaling pathways, promoting long-term survival and capacity for neurotrophic factor secretion [158]. Dhandapani et al. showed that E2 rapidly induces PI3K/Akt signaling in cultured astrocytes while significantly upregulating TGF-β1 and TGF-β2 protein expression [159]. Furthermore, E2-induced increases in Ca²^+^ in astrocytes predominantly arise from intracellular stores, which plays a key role in integrating synaptic signaling with glial function [160]. GPER-induced intracellular signalling promotes astrocytic formation of autophagosome, reduces pro-inflammatory cytokine release, and modulates excessive astrocyte activation caused by glutamate, thereby providing neuroprotection through MAPK signalling [161]. In ischemic models, E2 also protects astrocyte mitochondrial function via ER-dependent mechanisms [162].

Microglia and Neuroinflammation: GPER is highly expressed in activated microglia and is a crucial mediator of estrogen's anti-inflammatory effects. Both E2 and its selective agonist, G-1 cause a shift in microglial polarization from pro-inflammatory (M1) to anti-inflammatory (M2) phenotypes decreasing the release of pro-inflammatory cytokines such as TNF-α, IL-1β and IL-6 [163-165]. Moreover, following brain injury, E2 increases neurotrophic and anti-inflammatory factors, such as TGF-β and IGF-1, promoting tissue repair and neurogenesis [165].

Endothelial Function and Vascular Protection: One of the primary mechanisms by which E2 restores NVU integrity is through its regulation of endothelial cell function. Three main mechanisms are involved: Endogenous NO production: E2 causes a rapid increase in cGMP levels and increased NO production in cerebral microvessels [166-167]. Studies in vitro indicate that E2 activates eNOS via classical ER dependent PI3K/Akt phosphorylation pathways [168]. Confocal imaging demonstrates that ERα co-localizes with caveolin-1 in endothelial membrane caveolae, and disrupting caveolae abrogates E2-induced NO synthesis, underscoring the importance of membrane ERs in vascular protection [169].

GPER-mediated signaling: GPER is found on plasma membranes and within the endoplasmic reticulum of endothelial cells. Binding of E2 to GPER stimulates G protein-coupled signaling resulting in cAMP, Bcl-2, NGF, Cyclin D2 and c-Fos expression. GPER triggered heparinase-dependent EGF release activating EGFR transactivation, mobilizing intracellular Ca²^+^ and activating ERK1/2, Src and PI3K, which would lead to increased endothelial cell proliferation, increased NO production and opening K^+^ channels required for pressurizing cerebral microcirculation [170-171]. Anti-inflammatory and adhesion molecule suppression: E2 induces a downregulation in endothelial ICAM-1 decreasing the degree of leukocyte adhesion and dampening immune activation at the vascular interface [172-173]. Furthermore, in endothelial cells, E2 dampens excessive NF-κB pathways activation, possibly through IκB-α stabilization and other estrogen sensitive pathways [173].

Pericyte Function and Migration: Pericytes have ERα, ERβ and GPER and can respond to E2 signaling in a multitude of ways. E2 can inhibit TNF-α induced pericyte migration by ER-mediated inhibition of MAPK and Akt phosphorylation pathways which would maintain BBB integrity [174]. E2 can also regulate expression of anti-migratory and pro-migratory genes through transcriptomic analysis in pericytes [174]. Furthermore, downregulation of miR-638 through an ERβ mediated mechanism has been shown to prevent TNF-α induced pericyte migration [175].

In summary, E2 has multi-target effects across all NVU components enjoying positive neuronal effects, glial modulation, protection to endothelial cells, Pericyte migration, reduction of inflammation and repair of BBB integrity. Much work is needed to establish treatment windows, routes of delivery, and safety profiles to be able to elevate estrogen treatment to the advancement of clinical strategies for neurovascular restoration.

### 7.3 Mesenchymal Stem Cells and Exosomes

Mesenchymal stem cells (MSCs) have emerged as a promising cell-based therapy for AD and VD due to their low immunogenicity, ease of isolation from bone marrow or adipose tissue, and lower tumorigenic risk compared to induced pluripotent stem cells or neural stem cells [176]. MSCs exhibit multifaceted neurovascular protective properties through the secretion of a diverse array of neurotrophic factors (e.g., BDNF, GDNF, NGF, VEGF, and HGF) and regulatory miRNAs that together promote neuronal survival, neurogenesis, synaptic plasticity, and angiogenesis. These processes support NVU remodeling and restore brain homeostasis [177-178]. In addition, MSCs influence microglial activation to inhibit the M1 pro-inflammatory phenotype and generate the M2 anti-inflammatory phenotype, which ultimately leads to a reduction in chronic neuroinflammation and potentiates Aβ clearance through enhanced microglial autophagic activity around amyloid plaques [179].

Importantly, MSCs and their exosomes can demonstrate dynamic plasticity towards NVU pathology at each stage of AD, making the use of MSCs and MSC-exo as potential therapies an exciting field of research. In the early and middle stages, MSCs influence APP processing, inhibit Aβ production, influence Aβ degradation, reduce Tau hyperphosphorylation, and control proteasome activity [180]. In late stages of the disease, the effects of MSCs are primarily an action of microglial reprogramming, reduction of neuro-inflammation, and enhancement of antioxidative capacity [176]. Notably, these adaptive effects encompass therapeutic activities not only of MSCs themselves, but also of exosomes secreted by MSCs (MSC-exo) which are emerging as critical effectors of MSC-based therapies.

The neuroprotective therapeutic activity conferred on MSC-exo is highly dependent on the pathological environment. For example, hypoxia-preconditioned MSC-exo have exhibited superior efficacy in AD mouse models, including increased learning and memory, lower Aβ burden, increased synaptic protein expression, and suppressed neuroinflammation [181]. In contrast, exosomes derived from unconditioned MSCs demonstrated little neuroprotective therapeutic activity compared to MSC-exo from conditioned non-sourcederived stem cells, indicating that environmental factors impact the reparative profile of vesicles secreted from MSCs. The findings support the possibility of MSCs and MSC-exo providing context sensitive and stage adapted support of NVU integrity.

In summary, there is considerable evidence indicating multiple and synergistic effects of MSCs on NVU repair and functional maintenance based on paracrine secretion of neurotrophic factors and exosomes. These effects have great potential for further delaying or even reversing AD progression. However, there is still a lack of real-time monitoring of NVU functional parameters (e.g. dynamic contrast-enhanced MRI of cerebral blood flow and BBB permeability) or fluid biomarkers. Although a few small clinical trials have shown that MSC transplantation is safe, we have seen little cognitive improvement in AD patients, which may be due to less-than-optimal delivery routes or inability of cells to efficiently home to their targets [182]. As such, current research is aimed at improving administration routes, targeting delivery, and preconditioning approaches to further exploit the therapeutic effects of MSCs and exosomes in NVU restoration.

### 7.4 Remote Ischemic Conditioning

RIC is a simple, cost-efficient, non-invasive technique characterizing therapeutic, transient episodes of limb ischemia-reperfusion to activate endogenous protective mechanisms. Increasing evidence supports the use of RIC to elicit broad neurovascular protective effects that target multiple components of the NVU, making it a promising strategy for cerebrovascular diseases and related cognitive decline.

Vascular repair: One of the primary mechanisms by which RIC supports the brain is by promoting arteriogenesis and angiogenesis. Using a tMCAO mouse model, mice subjected to 14 consecutive days of RIC experienced a significant increase in cerebral blood flow, an increase in the diameter of the pial arteries, and an increase in vascular smooth muscle cell proliferation, with increases in Notch1 and the intracellular domain of Notch1 [183]. In animal models of vascular cognitive impairment (VCI), RIC stimulates angiogenesis via activation of the eNOS/NO/nitrite pathway [184]. The nitrite, which serves as a reservoir of NO, is elevated on reperfusion, via reactive hyperemia, to promote endothelial NO generation [185]. In fact, a single RIC session improved cerebral autoregulation for at least 24 hours in healthy individuals [186]. Clinically, RIC improves endothelial function in stroke survivors, as evidenced by increased flow-mediated dilation, an effect partly mediated by local GLP-1 signaling [187-188].

Neuroprotection: RIC protects neurons from ischemia-induced cell death through anti-apoptotic mechanisms while promoting pro-autophagic mechanisms. In BCAO models, RIC increases the expression of the cell survival gene Bcl-2 in the hippocampus and decreases neurodegeneration, helping to maintain cognition [189]. Stimulation of the JAK2-STAT3 and PI3K-Akt signaling pathways is likely responsible for the protective cellular mechanisms [190]. In addition, RIC also promotes autophagy by activating the expression of Beclin-1, LC3, Atg5-Atg12, Atg7 and decreasing p62, thereby promoting white matter protection and supporting hippocampal health [191].

Antioxidant and Anti-Inflammatory: RIC has also been shown to be an effective antioxidant intervention for vascular cognitive impairment. Applying RIC after bilateral common carotid artery occlusion reduces the ROS product such as malondialdehyde (MDA) and protects glutathione (GSH) levels in rats [192]. RIC has also been shown to enhance cognitive performance, increase the expression of HO-1 and GSH, and decrease MDA, neuroinflammation and hippocampal neurodegeneration in another rat study [193]. Mechanistically, RIC acts through the ET-1/hydrogen sulfide/Nrf2 pathway, activating the Nrf2 pathway and promoting oxidative stress defense [194]. In a study examining elderly patients (80-95 years of age) with symptomatic intracranial atherosclerosis (sICAS), RIC decreased systemic inflammatory markers (hsCRP, IL-6, leukocyte numbers and platelet aggregation) after30 days of intervention [195]. Lastly, in VCI mouse models, 2 weeks of RIC reduced the expression of ICAM-1, VCAM-1, GFAP and IBA-1 in the brain and reduced peripheral leukocyte adhesion and neuroinflammation [196]. These effects were associated with improved cognition, enhanced cerebral perfusion, reduced white matter damage, and attenuated Aβ accumulation.

BBB Integrity: RIC preserves BBB integrity, reduction in leakage is likely a result of suppressing the overactivation of MMP-9 and preserving tight junction proteins such as occludin and claudin-5 to reduce BBB permeability and cerebral edema due to reperfusion [197].

Through the availability of several protective mechanisms, specifically increases in cerebral perfusion, suppression of apoptosis and inflammation, stimulation of autophagy and preservation of BBB, RIC offers NVU protection as one comprehensive method of care. Its non-pharmacological, cost-effective nature and ease of administration make it a highly promising endogenous neuroprotective strategy for preventing and treating cerebrovascular diseases and cognitive decline. Further investigation is needed to understand the underlying mechanisms of activity, develop treatment protocols and wearable application methods to advance RIC for clinical considerations.

### 7.5 Mindfulness Meditation (MM)

Mindfulness meditation (MM) is a form of intentional, non-judgmental awareness practice that directs attention to present-moment experiences, aiming to cultivate inner calm and heightened self-awareness. There is a growing body of evidence supporting substantial benefits of MM for several neurocognitive disorders. Research has shown that MM can promote neuroplasticity, increase cortical thickness, reduce amygdala hyperreactivity and improve brain network connectivity and neurotransmitter balance, improving emotional coping, cognition, and resilience to stress [198]. Among older individuals, practice with MM has shown a positive association with memory, executive function, and information processing speed, which advocates for MM as a possible way to increase cognitive reserve [199-200]. Moreover, emotional dysregulation is shown to accelerate the course of dementia [201]. A randomized controlled study established that older adults who practiced MM were able to significantly reduce the rate of age-related brain volume loss [202].

Importantly, MM may promote recovery of the NVU and neurovascular coupling through modulations in the default mode network (DMN). MM practice improves the efficiency of the DMN and cognitive outcomes related to DMN for older adults [203]. Functional imaging studies demonstrated that MM increased the CBF at resting-state in the DMN and prefrontal cortex (PFC) regions in adults. These changes are thought to result from enhanced endothelial function and improved cellular metabolism, possibly mediated by MM-induced epigenetic upregulation of BDNF and eNOS gene expression through DNA methylation mechanisms [204-206]. MM also changes many metabolites in the brain, for example, intracellular pH, inorganic phosphate, and ATP product ratios, which suggests a remodeling of cerebral energy metabolism [207]. Specifically, greater resting-state functional connectivity (rsFC) within the DMN, after a training period in MM practice, was associated with reduced pro-inflammatory biomarkers [208]. Some evidence also suggests that MM may directly modulate central inflammatory signaling and glial cell activation through epigenetic mechanisms [204].

While clear molecular mechanisms are still not entirely clear, MM-induced activation of the PFC requires a stronger top-down cognitive control effect. Top-down cognitive control may be positive downstream on neurovascular health by reducing stress and emotional dysregulation and positively supporting neurovascular coupling pathways and cardiovascular regulation. In summary, MM offers an excellent "whole-system" intervention that may contribute to improving the protection and recovery of the NVU. Current research challenges include standardizing the implementation and assessment of this intentional mental practice to improve mechanistic certainty and clinical reproducibility. Due to the shortcomings of animal models in modeling any cognitive or complex behavior of humans, developing novel methodologies of evaluation and research will be very important.

## 8. Chinese Wisdom and Future Potential

After thousands of years of development, Chinese civilization has accumulated rich healthcare concepts and continuously improved them in practice. The unique life and disease perspectives of traditional Chinese medicine provide a holistic and dynamic approach to addressing health issues. In recent years, numerous studies have shown that many traditional therapies have positive effects in the treatment of cognitive impairments and brain injuries. This section will focus on introducing several traditional Chinese medicines and health care methods with potential for improving the neural vascular unit (NVU) and clarify their unique value in modern neurovascular protection.

### 8.1 Traditional Chinese Herbal Formula

Throughout the thousands of years of clinical practice and development, traditional Chinese herbal formula (TCHFs) has become the foundation of Traditional Chinese Medicine (TCM). Much different than Western single-target pharmacological approaches, these multi-component, multi-target, multi-pathway approaches provide a complementary therapeutic strategy. TCHFs amplify the additive effects of a number of herbs while at the same time offsetting specific herbal side effects through the rationality of the herbs combined. Despite complex mechanistic explanations of compound interactions that have yet to be clarified, principles for TCHF formulations have shown reliable safety and effectiveness when utilized for the re-balancing of multisystem disorders [209]. This value is particularly relevant for application in the treatment of degenerative nerve diseases according to TCM practice.

To illustrate, a number of TCHFs apply the low-competitive pharmacodynamics of compounds derived from plants. For instance, lignans, found in different herbs and classified as phytoestrogens, require gut microbiota (i.e., Bacteroides, Lactobacillus) to be biotransformed in order to bioactively generate compounds, enterodiol and enterolactone. These metabolites have been reported to exert bi-directional modulation of the estrogen receptors (active under low estrogen conditions, then becoming competitive to the receptor with hyper-expressed estrogen), representing an intrinsic advantage of botanical therapies [210]. The goal of combining such weak but complementary components is to amplify their cumulative therapeutic effects while reducing toxicity. The framework of "mild intervention + synergistic orchestration" is appropriately aligned with the NVU's complicated, multi-dimensional structural and pathological nature and enables more precise targeting of each component as needed However, there is still inadequate mechanistic research surrounding the interaction between the components of herbal formulas and the regulation of each component in a network, which inhibits scientific understanding and global acceptance.

Meta-analyses have provided empirical justification for the use of TCHFs in treating AD. In one systematic review, the authors reported significant cognitive improvements on the MMSE and ADAS-Cog scales (but mixed results with respect to daily function and neuropsychiatric symptoms) among patients with AD who were prescribed TCHFs [211]. In another study, both TCHF and TCHF plus donepezil, resulted in improvements in cognitive outcomes and reductions in plasma Aβ levels, suggesting a neuroprotective and possible disease modifying effect [212].

The effects of TCHFs can also be attributed to the synergistic pharmacologic effects of the herbs that constitute the formula. A comprehensive review provided evidence of TCM-derived active ingredients altering the NVU through enhancement of neuronal activity, repair of endothelial cells, inhibition of inflammation and oxidative stress, stabilization of the blood-brain barrier, and inhibition of hyperactivation of microglia [213]. While long-term use of multi-herb formulas raises concerns about potential side effects, this particular combination has a historically proven safety profile, underpinned by a well-established framework of compositional principles.

Recent research utilizing network pharmacology, multi-omics, and systems biology has begun to explain, in part, the pharmacologic mechanisms behind TCHFs so-called "multi-component-multi-target-multi-pathway" pharmacological effects. For example, Linggui Zhugan Decoction, was found through network analysis and animal studies, to modulate multiple signaling pathways while ameliorating behavior and pathology related to AD [214]. The therapeutic mechanisms of Dihuang Yinzi also implicate NVU modulation [215]. Spatial metabolomics analysis has shown that the use of the classic pairing of Schisandra chinensis and Panax ginseng affects at least 22 metabolites correlating to neuroinflammation, neurotransmitter imbalance, energy deficiency, and oxidative stress in AD [216]. Additionally, this combination enhances BBB permeability and compound stability, with polysaccharides from Schisandra improving the bioavailability of saponins and lignans. Another study addressed the pairing of Schisandra with Alpinia oxyphylla and observed significant reduction in the clearance rates of key active compounds suggesting prolonged systemic exposure and improved clinical efficacy in the management of cognitive disorders [217].

In summary, TCHFs offer a multi-target therapeutic potential with regards to NVU modulation, AD pathology and cognitive decline. They act on a greater but less specific range of biological targets than Western monotherapies, particularly involving glial-neurovascular-inflammation interactions. However, there are still limitations with regards to TCHFs, such as no standardized formulations and dosing recommendations, heterogeneity in clinical trials, and relatively few high-quality RCTs. Understanding the complex relationships between constituents has made pharmacokinetic modeling and safety assessments more complicated. While TCHFs are used in clinical practice in China for AD and VD, and their safety has been supported by network pharmacology and toxicology studies, our understanding of individualized prescriptions for TCHFs is still nascent. We have yet to learn whether a "golden formula" for the NVU is achievable and more research is needed to either discover and validate that formula or refute their credibility based upon rigorous evidence. Critically, new theories and experimental methodologies that integrate modern biomedical insights with traditional TCM logic are urgently required.

To support the internationalization and scientific standardization of TCHFs, future research should focus on:(1) Identifying core active compounds and their mechanisms of action;(2) Establishing quantifiable, reproducible metrics for quality and efficacy assessment;(3) Using omics technologies and AI to optimize formulation strategies;(4) Conducting large-scale, high-quality randomized controlled trials to validate their multi-target efficacy and safety in AD and VD.

### 8.2 Traditional Chinese Exercises

Traditional Chinese exercises, such as Tai Chi, Baduanjin, and Qigong, are foundational health-preserving practices in TCM, which embodies the philosophy of "mind-body unity and dynamic balance." These practices emphasize the coordinated integration of breath, movement, and intention to maintain overall well-being. They practice traditionally combine slow and gentle movement with a rhythmic quality of breathing and awareness, essentially combining aerobic exercise, resistance training, fine-motor control, dynamic balance, meditative practice, and memory training, all of which is achieved in one multi-faceted structure. This multifaceted structure offers a holistic approach to physical and mental health regulation.

While the complexity and culture-specific nature of traditional exercises limits their uniformity in preclinical animal models, increasing numbers of clinical trials are examining the beneficial effects of traditional exercises in patients with cognitive impairment. A recent systematic review and meta-analysis demonstrated significant cognitive improvements in individuals with MCI following traditional exercise interventions, as reflected by improvements in the Montreal Cognitive Assessment (MoCA) (SMD = 1. 04, 95% CI: 0. 71-1. 38) and Mini-Mental State Examination (MMSE) (SMD = 0. 82, 95% CI: 0. 41-1. 42) scores [218]. A further subgroup analysis showed improvements in multiple cognitive areas, including language function, executive function, short-term memory, and delayed recall [219]. Furthermore, traditional exercises can improve sleep quality, decrease mood disturbances, and lower blood lipids [220-221], all concepts that are indirectly related to Alzheimer's disease (AD) pathology. Notably, several trials demonstrated increased BDNF expression following traditional exercise in older adult populations with cognitive decline and can imply increases in neuroplasticity or neuroprotection [222]. In addition, traditional exercises may complement pharmacological treatments resulting in augmented effects of both treatments [223].

These findings are not unexpected. Many studies have reported that regular resistance training and mindfulness meditation will improve NVU function, as stated earlier in Section 6. Similarly, there is increasing evidence that cognitive training contributes to the maintenance and restoration of brain functioning in neurodegenerative diseases [224]. This evidence suggests that traditional forms of physical and mental exercise may have unique benefits to prevent or intervene with the repair of the neurovascular unit and neurodegenerative diseases. They are inducing similar effects as resistance training and meditation, through a multi-level coordinated regulation of cerebral blood flow perfusion, neurotrophic, immune-inflammatory and neural homeostasis. In other words, traditional mind-body exercises provide a Chinese-style solution that integrates multiple effective self-training approaches, serving the systemic reconstruction of NVU.

However, the current literature has multiple limitations. First, the body of literature lacks standardized, and systematic intervention protocols designed specifically for cognitive impairment. Current research has several limitations. Second, due to the complex and variable nature of traditional exercise routines, study designs face challenges, with few studies exploring the underlying mechanisms or tracking biological markers to elucidate causality. Third, much of the literature is based on small RCTs that lack duration to examine the longer-term benefits, survival of effects or specific effects among high-risk populations of individuals including those individuals with MCI.

Future research should focus on the following areas: (1) Clearly define appropriate exercise types, intensity, frequency, and duration for specific populations, and establish standardized movement-based interventions for patients with AD and MCI. (2) Utilize neuroimaging, physiological monitoring, and neurobiomarker assays to visualize and quantify the effects of traditional exercise interventions. (3) Investigate potential mechanisms based on modern biomedical theories and explore synergistic effects with pharmacological and nutritional therapies. (4) Systematically assess the efficacy, response variability, and underlying mechanisms of traditional Chinese exercises across different stages of cognitive impairment.

### 8.3 Acupuncture and Related Therapies

As a widely used non-pharmacological intervention in TCM, acupuncture has gained attention for its simplicity, high safety profile, and minimal adverse effects. It has been extensively applied in the management of neurodegenerative disorders such as AD, VD and cognitive impairment. In recent years, growing clinical and preclinical evidence has supported its therapeutic efficacy. A meta-analysis showed that acupuncture significantly improved cognitive function and quality of life in patients with AD, with a favorable safety profile [225]. Another systematic review that included 15 randomized controlled trials (RCTs) of acupuncture for amnestic mild cognitive impairment (aMCI) indicated potential cognitive benefits, though high-quality, large-sample studies are still needed to confirm its efficacy [226].

As a core TCM intervention, acupuncture modulates multiple systems by stimulating specific acupoints, embodying the TCM philosophy of "multi-target, multi-level regulation. " This aligns well with the complex pathophysiology of AD and VCI, both of which involve synergistic dysfunction across the NVU. Increasing studies have revealed that acupuncture can modulate NVU components on multiple levels. A review article elaborated that acupoint stimulation can: (1) modulate glutamate and acetylcholine levels as well as the activity of their metabolic enzymes; (2) inhibit inflammatory responses in brain tissue and eliminate free radicals; (3) enhance antioxidant capacity, reduce neuronal apoptosis, and regulate the expression of microtubule-associated proteins. These effects together improve synaptic function and neural network structure, thereby ameliorating cognitive status [227].

The effects of acupuncture vary by acupoint. Stimulation of Baihui (GV20), Dazhui (GV14), and Mingmen (GV4) was reported to reduce nitric oxide (NO) and malondialdehyde (MDA) levels, elevate superoxide dismutase (SOD) activity, and alleviate oxidative stress, thereby preserving neurons and microvasculature in aging mouse brains [228]. In AD models, acupuncture at GV14, Shenshu (BL23), and Taixi (KI3) downregulated pro-inflammatory cytokines IL-1 and IL-6, suggesting inhibition of microglial overactivation and mitigation of NVU damage [229]. Electroacupuncture (EA) at Yongquan (KI1) improved monoamine neurotransmitter levels, neural network integrity, and neurovascular coupling [230]. Separately, EA at Baihui modulated cerebral metabolites (e.g., NAA, Glu, mI), reducing excitotoxicity-induced neuronal and BBB injury [231].

Combination therapies have demonstrated synergistic influence. For example, electrical EA at Baihui and Yintang improved the cognitive effects of donepezil, potentially by facilitating transport across the BBB and regulating the MMP-9, LRP1 and Aβ metabolic pathways, which would allow for improved Aβ clearance across the NVU [232]. Alternating stimulation at KI1 increased hippocampal acetylcholinesterase activity and improved cholinergic functioning [233]. Acupuncture at GV20, Fengfu (GV16), BL23, and Xuanzhong (GB39) improved cognitive performance, improved total antioxidant capacity (T-AOC), and decreased MDA, further supporting its multi-targeted effects on immune modulation and BBB stabilization [234].

Novel studies have also utilized functional imaging techniques such as fMRI and near-infrared spectroscopy (NIRS) to measure neural circuits and brain activation patterns influenced by acupuncture [235-236]. While human studies are inconclusive regarding the effects of acupuncture on CBF, animal studies have demonstrated that acupuncture can activate CBF-regulating mechanisms and lead to improvements in cognition [237-238].

However, there are several limitations: (1) inconsistencies in acupoint selection, stimulation protocols, and outcome measures hinder reproducibility; (2) most clinical trials are small-scale, lacking multicenter validation and long-term follow-up; (3) mechanistic studies remain largely preclinical, with limited integration of objective biomarkers and functional assessments.

It should be noted that one of the distinctive benefits of acupuncture is its flexibility to individualized treatment. In addition to modulating NVU structures and functions, acupuncture may also regulate comorbid risk factors such as hypertension [239] and metabolic disturbances [240], offering a holistic strategy for cognitive intervention. Future studies should focus on: (1) clarifying the neural pathways and targets of individual acupoints based on contemporary neuroanatomy while also conceptualizing function-anatomy-based acupoint combinations focused on specific types of NVU dysfunction; (2) developing protocols for standardized interventions; (3) incorporating omics, imaging, and multimodal monitoring technologies to measure NVU changes; and (4) conducting high-quality randomized controlled trials with large samples and multi-center designs to support clinical translation.

## 9. Conclusion and Perspectives

As we grow to better understand the pathogenesis of cognitive disorders, the NVU has emerged as a pivotal regulatory hub in maintaining central nervous system homeostasis. Its pathological remodeling in AD and VD has drawn growing attention. In this review, we discuss the common neurovascular mechanisms contributing to both AD and VD and illustrate the progressive disruption of the NVU during the progression of the disease, and the current multi-target and multi-modal approaches to promote NVU restoration.

Collectively, the concept of the NVU appears to provide a unified framework to link the pathological processes and mechanisms that underpin the various forms of progressive dementia. This approach offers an innovative paradigm for understanding and designing dementia interventions.

Nowadays, interventions such as physical exercise, estrogen administration, stem cell therapies, and TCM are all increasing evidence of the ability to NVU function through multi-level and multi-mechanical pathways, and this opens up new opportunities in the prevention and treatment of cognitive impairment. TCM techniques are characterized by integrated methods, (i.e., herbal formulae, mind-body practices, and acupoint stimulation) that offer opportunities for personalized interventions in response to the complex and multifactorial pathology of NVU dysfunction. That being said, there remains a substantial challenge in identifying, determining and optimizing the optimal combination of TCM techniques, which require further rigorous validation.

Future NVU research should focus on: (1) To investigate the connections and regulatory interactions among NVU components, as well as their associations with risk factors and disease states; (2) To validate the mechanisms of interventions targeting NVU using imaging, biomarkers, and other monitoring; (3) To develop clinical protocols and opportunities for combinatory applications based on the properties of NVU-directed interventions; (4) To explore NVU-targeted approaches to uncover potential synergistic effects with single-target pharmacotherapy, including preliminary indications and standardization; (5) To identify TCM-based multi-component interventions with potential for modern translational application.

In the future, interdisciplinary collaboration and integration of Western and traditional medicine will be essential for advancing precision interventions for cognitive disorders.
